# Total Burden of Cerebral Small Vessel Disease in Recurrent ICH versus First-ever ICH

**DOI:** 10.14336/AD.2018.0804

**Published:** 2019-06-01

**Authors:** Mangmang Xu, Yajun Cheng, Quhong Song, Ruozhen Yuan, Shuting Zhang, Zilong Hao, Ming Liu

**Affiliations:** Center of Cerebrovascular Disease, Department of Neurology, West China Hospital, Sichuan University.Chengdu, 610041, Sichuan Province, China

**Keywords:** recurrent intracerebral hemorrhage, cerebral small vessel disease, primary intracerebral hemorrhage, white matter hyperintensity, cerebral microbleed

## Abstract

The relationship between recurrent intracerebral hemorrhage (ICH) and total burden of cerebral small vessel disease (CSVD) is not completely investigated. We aimed to study whether recurrent intracerebral hemorrhage (ICH) had higher CSVD score than first-ever ICH. Lacunes, white matter hyperintensities (WMH), cerebral microbleeds (CMBs), enlarged perivascular spaces (EPVS), cortical superficial siderosis (cSS) and CSVD score were rated on brain magnetic resonance imaging (MRI) in primary ICH patients. Recurrent ICHs were confirmed by reviewing the medical records and MRI scans. Mixed hematomas were defined as follows: deep + lobar, deep + cerebellar, or deep + lobar + cerebellar. Of the 184 patients with primary ICH enrolled (mean age, 61.0 years; 75.5% men), recurrent ICH was present in 45 (24.5%) patients; 26.1% (48/184) had ≥2 hematomas, 93.8% (45/48) of which exhibited recurrent ICH. Mixed hematomas were identified in 8.7% (16/184) of patients and bilateral hematomas in 17.9% (33/184). All mixed hematomas and bilateral hematomas were from cases of recurrent ICH. Patients with mixed etiology-ICH were more likely to have recurrent ICH than patients with cerebral amyloid angiopathy (CAA) or hypertensive angiopathy (HA)-related ICH (36.8% vs17.8%, *p*=0.008). Multivariate ordinal regression analysis showed that the presence of recurrent ICH (*p*=0.001), ≥2 hematomas (*p*=0.002), mixed hematomas (*p*<0.00001), and bilateral hematomas (*p*=0.002) were separately significantly associated with a high CSVD score. Recurrent ICH occurs mostly among patients with mixed etiology-ICH and is associated with a higher CSVD burden than first-ever ICH, which needs to be verified by future larger studies.

Original Article

Cerebral small vessel disease (CSVD) indicates a group of pathological processes acting on the arterioles, capillaries and venules of the brain and resulting in both ischemic and hemorrhagic lesions [1, 2]. CSVD is a very common cause of substantial cognitive [3] and psychiatric [4] disabilities in older people, and contributes to 45% of dementias [5].

On brain magnetic resonance imaging (MRI), four closely correlated manifestations including lacunes, cerebral microbleeds (CMBs), white matter hyperintensities (WMH), enlarged perivascular spaces (EPVS) and cortical superficial siderosis (cSS) are markers of CSVD [2, 6, 7]. However, one patient may present these markers simultaneously [8]. Therefore, the total burden of CSVD might better capture the overall effect of CSVD than only one or two individual markers [9]. Recently, one total CSVD score was proposed [9-12] to reflect the overall effect of CSVD on the brain.

Primary intracerebral hemorrhage (ICH), which mainly consists of cerebral amyloid angiopathy (CAA) and hypertensive angiopathy (HA)-related ICH [2], is supposedly due to small vessel disease [13]. One longitudinal study enrolling heterogeneous cohorts without specifically focusing on ICH [14] reported that recurrent stroke is associated with severe CSVD. In addition, a recent review and meta-analysis demonstrated that multiple CMBs were associated with high ICH recurrence [15, 16]. However, to the best of our knowledge, whether the ICH event influences CSVD progression is unclear. Therefore, in this cross-sectional study, we sought to assess (1) the burden and distribution of individual CSVD markers and (2) compare the total burden of CSVD using the reported CSVD score in those patients with recurrent ICH with the total burden in those with first-ever ICH in the setting of primary ICH.

## MATERIALS AND METHODS

### Study population

A consecutive cohort of ICH patients with MRI brain examination who were admitted to the Department of Neurology or Neurosurgery, West China Hospital, Sichuan University from January 2012 to June 2017 were retrospectively reviewed. Patients were encluded in the present study if they had primary ICH and underwent brain MRI including susceptibility weighted imaging (SWI) sequence during hospitalization. Patients with (1) secondary ICH such as aneurysm, arteriovenous malformation, Moyamoya disease, medication or tumor-related ICH, or hemorrhagic transformation after ischemic stroke; (2) primary intraventricular hemorrhage (IVH); (3) a history of ischemic stroke; or (4) poor image quality were excluded. This study was approved by The Medical Ethics Committee of West China Hospital. Informed consent was obtained from all included patients or next-of-kin.

### Data collection

Demographic information and baseline characteristics including age; sex; time from ICH onset to MRI scan, and comorbid conditions, including history of hypertension, diabetes mellitus, hyperlipidemia, history of ICH, and smoking, were retrieved.

Recurrent ICH was defined according to ICH history from the medical records and by reviewing participant’s brain MRI-SWI scan, which was sensitive to old hemorrhage as well as recent hemorrhage. Hematoma location was classified into deep [i.e., basal ganglia (BG), thalamus, brain stem], lobar (i.e., cortical-subcortical) or cerebellar. Both old ICHs and new ICHs were calculated in the number of hematomas. Mixed hematomas were defined as follows: deep + lobar, deep + cerebellar, or deep + lobar + cerebellar. Mixed CMBs were defined the same way as mixed hematomas. Bilateral hematomas were defined as hematomas in bilateral hemispheres. Patients ≥55 years of age with strictly lobar ICH involving cortical or cortico-subcortical regions (cerebellar ICH allowed) with or without pure lobar CMBs, but no deep CMBs were coded as cases of cerebral amyloid angiopathy (CAA)-ICH according to the modified Boston criteria[17]. Hypertensive angiopathy (HA)-ICH was defined when patients presented with pure deep ICH with or without deep CMBs but no lobar CMBs[18, 19]. Patients ≥55 years of age with both deep and lobar hematoma/CMBs were defined as having mixed etiology-ICH, while those <55 years of age with lobar ICH with or without deep hematoma/CMBs were defined as having undetermined etiology-ICH.

### MRI acquisition

All included patients underwent brain MRI (median 5.95 days, interquartile range 3.86 to 14.79 days after ICH onset) on a 3T MR scanner (Siemens; matrix size, 256 × 256 pixels). Sequences included fluid-attenuated inversion recovery (FLAIR) [repetition time (RT) 6000 ms; echo time (ET) 100 ms]; T1- (RT 1600 ms; ET 8.6 ms); T2-weighted image (RT 4500 ms, ET 105 ms); and SWI (RT 207 ms, ET 20 ms).

### Assessment of each CSVD marker

The MRI markers of CSVD were rated in line with STRIVE consensus criteria [13].

We defined lacunes as rounded or ovoid lesions of cerebral spinal fluid signal, that is, hyperintensities on a T2-weighted sequence with corresponding hypointensities with a hyperintense rim on FLAIR, ranging from 3 to 20 mm in diameter[8, 9], in bilateral hemispheres.

CMBs were defined as small (<10 mm in diameter), homogeneous, round lesions with low signal intensity on SWI in bilateral hemispheres and differentiated from mineral depositions and vessel flow voids according to the recent consensus [9, 20, 21].

Cortical superficial siderosis (cSS), the marker of CAA, was defined as curvilinear residues of chronic blood products following the cortical surface distinct from vessels and was detected as linear hypointensities on SWI [7, 22]. The severity of cSS was classified into disseminated (involving >3 sulci) or focal (≤3 sulci) in line with previous studies [7, 22].

WMH were assessed on T2-weighted and FLAIR images in deep and periventricular regions in the ICH-free hemisphere or in the hemisphere with the smaller hematoma when patients had bilateral hematomas. The severity of WMH was rated using the Fazekas scale from 0 to 3 [20, 23]. The presence of WMH was defined as deep WMH with a Fazekas score of 2-3 and/or periventricular WMH with a Fazekas score of 3 [9, 14]. The sum of the scores for deep and periventricular WMH was considered as the total WMH score [20].

**Table 1:** Clinical and neuroimaging characteristics of the study population.

Variable	All participants (n=184)	Recurrent ICH (n=45)	First-ever ICH (n=139)	*P* Value
Clinical characteristics				
Age, Y, mean (SD)	61.0 (12.5)	60.8 (10.4)	61.0 (13.1)	0.902
Sex, male, n (%)	139 (75.5)	30 (66.7)	109 (78.4)	0.111
History of hypertension, n (%)	122 (66.3)	34 (75.6)	88 (63.3)	0.131
History of DM, n (%)	17 (9.2)	2 (4.4)	15 (10.8)	0.250
History of hyperlipidemia, n (%)	6 (3.3)	3 (6.7)	3 (2.2)	0.158
Smoking, n (%)	61 (33.2)	10 (22.2)	51 (36.7)	0.073
ICH etiology				
CAA-ICH, n (%)	26 (14.1)	5 (11.1)	21 (15.1)	0.066
HA-ICH, n (%)	75 (40.8)	13 (28.9)	62 (44.6)	
Mixed etiology-ICH, n (%)	57 (31.0)	21 (46.7)	36 (25.9)	
Undetermined, n (%)	26 (14.1)	6 (13.3)	20 (14.4)	
Neuroimaging characteristics				
ICH location,				
Deep, n (%)	128 (69.6)	28 (62.2)	100 (71.9)	0.177
Lobar, n (%)	48 (26.1)	13 (28.9)	35 (25.2)	
Cerebellum, n (%)	8 (4.3)	4 (8.9)	4 (2.9)	
Presence of lacunes, n (%)	83 (45.1)	26 (57.8)	57 (41.0)	0.049
Presence of WMH, n (%)	90 (48.9)	33 (73.3)	57 (41.0)	<0.0001
The severity of WMH				
Total WMH 0-2, n (%)	81 (44.0)	10 (22.2)	71 (51.1)	0.002
Total WMH 3-4, n (%)	50 (27.2)	15 (33.3)	35 (25.2)	
Total WMH 5-6, n (%)	53 (28.8)	20 (44.4)	33 (23.7)	
Presence of CMBs, n (%)	135 (73.4)	40 (88.9)	95 (68.3)	0.007
The severity of CMBs,				
0, n (%)	49 (26.6)	5 (11.1)	44 (31.7)	0.014
1-4, n (%)	63 (34.2)	16 (35.6)	47 (33.8)	
≥5, n (%)	72 (39.1)	24 (53.3)	48 (34.5)	
Strictly deep CMBs, n (%)	45 (24.5)	11 (24.4)	34 (24.5)	0.998
Strictly lobar CMBs, n (%)	27 (14.7)	7 (15.6)	20 (14.4)	0.848
Mixed location CMBs, n (%)	63 (34.2)	22 (48.9)	41 (29.5)	0.017
The presence of cSS, n (%)	44 (23.9)	14 (31.1)	30 (21.6)	0.193
The presence of disseminated cSS, n (%)	20 (10.9)	6 (13.3)	14 (10.1)	0.584
Presence of EPVS>10 in BG, n (%)	92 (50.0)	23 (51.1)	69 (49.6)	0.864
The severity of EPVS in BG				
≤10, n (%)	92 (50.0)	22 (48.9)	70 (50.4)	0.686
11-20, n (%)	49 (26.6)	14 (31.1)	35 (25.2)	
>20, n (%)	43 (23.4)	9 (20.0)	34 (24.5)	
The severity of EPVS in CSO				
≤10, n (%)	78 (42.4)	15 (33.3)	63 (45.3)	0.069
11-20, n (%)	46 (25.00	9 (20.0)	37 (26.6)	
>20, n (%)	60 (32.6)	21 (46.7)	39 (28.1)	
CSVD score, median (IQR)	2 (1-3)	3 (2-4)	2 (1-3)	0.002

Abbreviation: ICH, intracerebral hemorrhage; DM, diabetes mellitus; CAA, cerebral amyloid angiopathy; HA, hypertensive angiopathy; CSVD, cerebral small vessel disease; CMBs, cerebral microbleeds; WMH, white matter hyperintensity; cSS, cortical superficial siderosis; EPVS, enlarged perivascular spaces; CSO, centrum semiovale; BG, basal ganglia; SD, standard deviation; IQR, interquartile range.

EPVS were defined as <3 mm punctate or linear hyperintensities on a T2-weighted sequence with corresponding hypointensities on a T1/FLAIR sequence [9, 20]. EPVS were rated in the centrum semiovale (CSO) and BG in the ICH-free hemisphere or in the hemisphere with the smaller hematoma when patients had bilateral hematomas. The number of EPVS referred to EPVS in the slice and side with the greatest extent of EPVS after review of all relevant slices of BG or CSO [24].

### Determining the overall burden of CSVD

Based on the recently proposed CSVD score [9], we assessed the total burden of CSVD on an ordinal scale ranging from 0 to 4 by allocating 1 point to each of the following: the presence of any lacune, any CMB, the presence of WMH, and the number of BG EPVS>10.

All CSVD markers and hematoma characteristics were assessed by two certified and experienced neurologists (M.X. and Y.C.) blinded to clinical information. Interrater agreement for the identification of CSVD makers and hematoma characteristics was good and even excellent with kappa ranged from 0.643 to 1.000. Kappa values of each imaging variable are shown in supplementary Table 1.

### Statistical analysis

Patients were divided into two groups based on the presence or absence of recurrent ICH. We compared the clinical information and imaging characteristics using Student t test or Mann-Whitney U test for continuous data, andχ^2^ test or Fisher exact test for categorical data when appropriate. Multivariate ordinal regression analysis with CSVD score as the dependent variable was performed to assess the association with the presence of recurrent ICH, ≥2 hematomas, mixed hematomas, and bilateral hematomas separately, by adjusting for age, sex, and risk factors (history of hypertension, diabetes mellitus, hyperlipidemia and smoking, which are frequently reported to be associated with individual CSVD markers [14]). Interrater reliability of neuroimaging variable was tested using the kappa statistic. All analyses were performed using SPSS software (version 21; IBM, New York, NY).

## RESULTS

### Population

**Figure 1.**
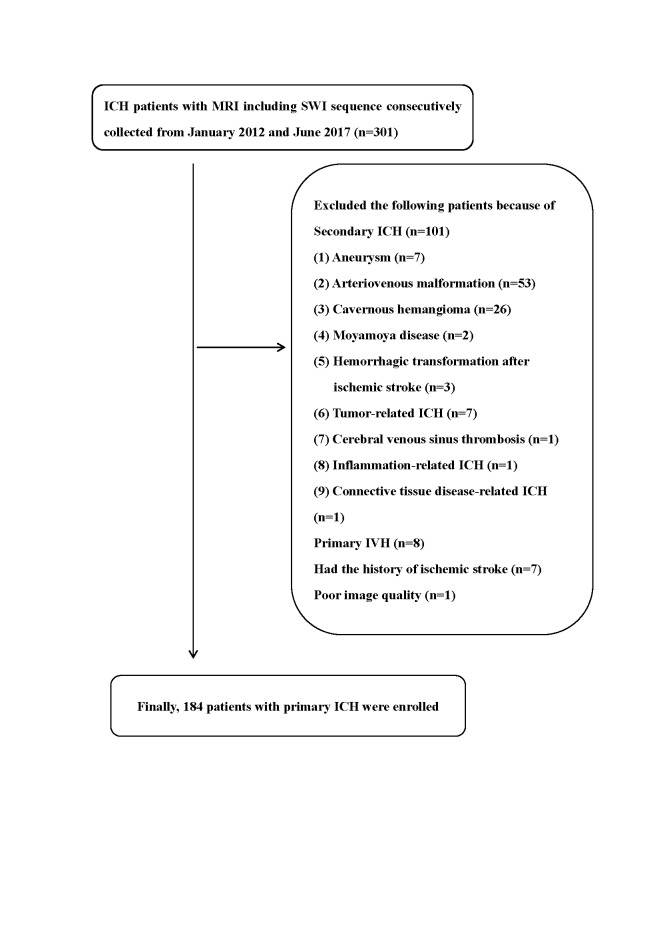
Flow gram of patient selection.

A total of 301 ICH participants with MRI-SWI were admitted to our center from January 2012 through June 2017. We excluded 101subjects with secondary causes, 8 with primary IVH, 7 with the history of ischemic stroke and 1 due to poor image quality. Thus, 184 participants with a diagnosis of primary ICH were included in the analysis (Fig. 1).

Of the included participants, their mean age was 61.0 years, and 75.5% were men. Overall, 26 (14.1%) had CAA-ICH, 75 (40.8%) had HA-ICH, 57 (31.0%) had mixed etiology-ICH and 26 (14.1%) had undetermined etiology-ICH. Recurrent ICH was identified in 45 (24.5%) participants. The clinical and neuroimaging characteristics of all included participants, and the subgroups with recurrent ICH and first-ever ICH are summarized in Table 1. There were no significant differences among the two groups with regard to demographic characteristics, comorbid conditions, ICH etiology, or ICH location.

Patients with CAA-ICH had a higher incidence of recurrence than those with HA-ICH [19.2% (5/26) versus (vs) 17.3% (13/75)] without reaching statistical significance. In contrast, patients with mixed etiology-ICH were more likely to have recurrent ICH than those with CAA- or HA-ICH (36.8% vs 17.8%, *p*=0.008, Pearson χ^2^ test). Supplementary Table 2 shows the severity of WMH by ICH etiology; the data show that there was a significant difference among patients with HA-, CAA-, and mixed etiology- ICH with regard to the presence of WMH (P<0.00000001), the presence of deep WMH with a score of 2-3 (P<0.0000001), and the presence of periventricular WMH with a score of 3 (P<0.00001). The presence of WMH, deep WMH with a score of 2-3 and periventricular WMH with a score of 3 was the highest in patients with mixed etiology-ICH, followed by those with CAA-ICH and HA-ICH.

**Table 2:** Total CSVD score for patients with recurrent ICH and first-ever ICH.

CSVD score	All participants (n=184)	Recurrent ICH (n=45)	First-ever ICH (n=139)
0, n (%)	25 (13.6)	2 (4.4)	23 (16.5)
1, n (%)	36 (19.6)	8 (17.8)	28 (20.1)
2, n (%)	41 (22.3)	8 (17.8)	33 (23.7)
3, n (%)	46 (25.0)	10 (22.2)	36 (25.9)
4, n (%)	36 (19.6)	17 (37.8)	19 (13.7)

Abbreviation: CSVD, cerebral small vessel disease; ICH, intracerebral hemorrhage

Overall, 48 (26.1%) patients had ≥2 hematomas and 11 (6.0%) had ≥3 hematomas among the 184 participants. Of those with ≥2 hematomas, 93.8% (45/48) exhibited recurrent ICH, 4.2% (2/48) exhibited first-ever ICH with multiple hematomas, and 2.1% (1/48) had two ICH events located in the same region. In contrast, all of the 11 participants with ≥3 hematomas had recurrent ICH. Mixed hematomas were identified in 16 (8.7%) participants. Of these, the most prevalent type was deep + lobar (68.8%, 11/16), followed by deep + cerebellar (18.8%, 3/16) and deep + lobar + cerebellar (12.5%, 2/16). Bilateral hematomas were found in 33 (17.9%, 33/184) participants. All of the patients with either mixed hematomas or bilateral hematomas exhibited recurrent ICH.

**Figure 2.**
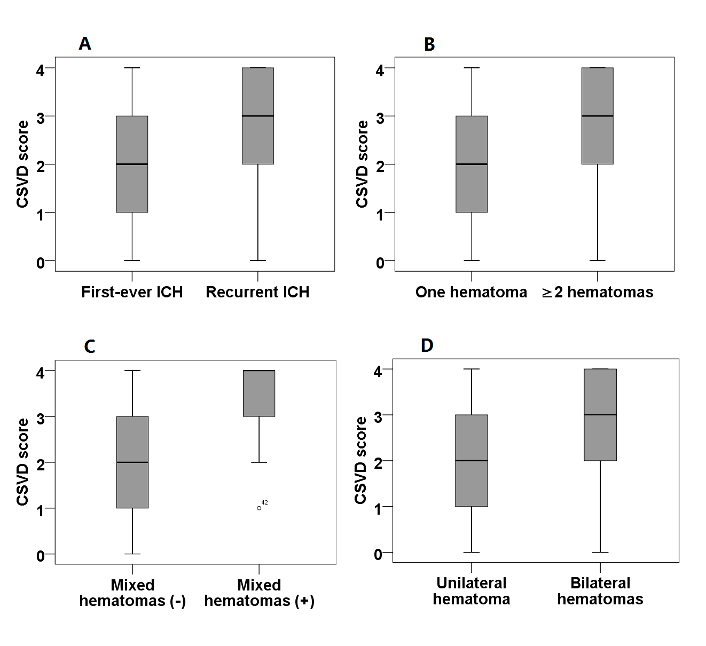
Hematoma characteristics and CSVD score. **A**) the presence of recurrent ICH and CSVD score. **B**) The number of hematomas and CSVD score. **C**) the presence of mixed hematomas and CSVD score. **D**) the presence of bilateral hematomas and CSVD score.

### Association between ICH characteristics and total CSVD score

Of the four CSVD markers, recurrent ICH was more often found in patients with lacunes (*p*=0.049), WMH (*p*<0.0001), and CMBs (*p*=0.007) (Table 1). As expected, recurrent ICH was associated with a higher CSVD score than first-ever ICH [Table 1, *p*=0.002 (Mann-Whitney test); Table 2, *p*=0.006 (Pearson χ^2^ test)] (Fig. 2A), mainly due to the higher prevalence of lacunes, WMH, and CMBs in the former. Meanwhile, the presence of recurrent ICH was significantly associated with an increasing burden of WMH (*p*=0.002), especially those with WMH with a score of 5-6. Similarly, recurrent ICH correlated with a high burden of CMBs (*p*=0.014). When stratifying participants by CMB location, recurrent ICH correlated with a high incidence of mixed CMBs (*p*=0.017), while strictly deep CMBs and strictly lobar CMBs did not differ between patients with recurrent ICH and first-ever ICH. For cSS and EPVS, there were no significant differences between recurrent and first-ever ICH with regard to the presence of cSS, the presence of disseminated cSS, BG EPVS>10, and the severity of BG EPVS and CSO EPVS.

In univariate analysis, the presence of recurrent ICH (*p*=0.001), ≥2 hematomas (*p*=0.001), mixed hematomas (*p*<0.00001), and bilateral hematomas (*p*=0.003) were all separately significantly associated with an increased CSVD burden (Table 3, Fig. 2 A-D). After fully adjusting for sex, age, history of hypertension, diabetes mellitus, hyperlipidemia, and smoking, the associations with the presence of recurrent ICH (*p*=0.001), ≥2 hematomas (*p*=0.002), mixed hematomas (*p*<0.00001), and bilateral hematomas (*p*=0.002) were still significant, especially the association with mixed hematoma.

### DISCUSSION

In the present study, we first showed that patients with mixed etiology-ICH more often had recurrent ICH than patients with CAA- or HA-ICH. In addition, we found that patients with recurrent ICH had a higher CSVD score than those with first-ever ICH, mainly due to a higher prevalence of lacunes, WMH, and CMBs in the former. Additionally, we reported that the presence of ≥2 hematomas, mixed hematomas, and bilateral hematomas were separately associated with a higher CSVD score.

Consistent with previous study [15], our data showed that patients with recurrent ICH were more likely to have CMBs and more severe CMBs than those with first-ever ICH. In the past year, the presence of high-grade WMH was also demonstrated to be correlated with stroke recurrence presenting as ICH, atherothrombotic infarctions, and lacunar infarctions in stroke patients [25], which we confirmed in our cohort with a specific focus on primary ICH. However, all former research focused on individual CSVD markers in MRI scans without evaluating the combination of markers as one disease [6] in the setting of primary ICH. Therefore, we compared the association of total burden of CSVD by combining the four markers in recurrent ICH versus that in first-ever ICH.

**Table 3:** Association between ICH and total burden of CSVD in ordinal regression analysis.

Variable	Unadjusted		Age- + sex-adjusted		Fully adjusted	
	OR (95% CI)	*p*	OR (95% CI)	*p*	OR (95% CI)	*p*
Recurrent ICH	0.361 (0.195 to 0.668)	0.001	0.313 (0.167 to 0.587)	<0.001	0.332 (0.174 to 0.632)	0.001
≥2 hematomas	0.375 (0.206 to 0.685)	0.001	0.334 (0.181 to 0.616)	<0.001	0.365 (0.195 to 0.683)	0.002
Mixed hematomas	0.093 (0.034 to 0.258)	<0.00001	0.064 (0.022 to 0.187)	<0.000001	0.073 (0.024 to 0.216)	<0.00001
Bilateral hematomas	0.354 (0.178 to 0.705)	0.003	0.288 (0.142 to 0.582)	0.001	0.322 (0.156 to 0.664)	0.002

Fully adjusted: age, sex, history of hypertension, diabetes mellitus, hyperlipidemia, and smoking Abbreviation: CSVD, cerebral small vessel disease; ICH, intracerebral hemorrhage; OR, odds ratio; CI, confidence interval

When focusing on the severity of WMH and ICH etiology, we found that mixed etiology-ICH had the highest proportion of patients exhibiting WMH, deep WMH with a score of 2-3, and periventricular WMH with a score of 3, followed by CAA-ICH and HA-ICH. Our results were in line with those in a previous study [26] that showed that patients with mixed ICH had the highest WMH volume when compared to those with HA-ICH and CAA-ICH.

Meanwhile, participants with ≥2 hematomas, mixed hematomas, and bilateral hematomas were also shown to have a higher CSVD score than those with one hematoma, non-mixed hematomas, and unilateral hematoma, respectively. The above associations were based on the association of recurrent ICH and total burden of CSVD, since recurrent ICH occurred in 93.9% among those with ≥2 hematomas, and all patients with mixed hematomas and bilateral hematomas exhibited recurring ICHs.

However, our study had several limitations. First, in the present study, 10 participants had bilateral BG hematoma, resulting in no hematoma-free BG to rate EPVS and periventricular WMH; thus, BG EPVS and periventricular WMH in the 10 patients were rated in the hemisphere with the smaller hematoma, potentially resulting in detection bias in the 10 participants. However, after excluding the 10 participants, the association of CSVD score with the presence of recurrent ICH (*p*=0.001), ≥2 hematomas (*p*=0.001), mixed hematomas (*p*<0.0001), and bilateral hematomas (*p*=0.001) separately still remained significant after fully adjusting. Second, not all ICH patients received MRI scans. Patients who were too sick, had contraindications of MRI examination, refused to undergo MRI, or those family members refused to allow MRI were excluded. In the study period, ICH in 1033 patients with CTA or DSA examination and without MRI (including SWI sequence) might be caused by HA or CAA. Data showed that those patients were younger and more likely to have hypertension. Furthermore, the condition of those patients was more severe (lower GCS and higher NIHSS) than the condition of the patients with SWI examination (see supplementary Table 3). However, there were not significant differences in hematoma location in patients with and without MRI. Thus, the results in this present study need to be interpreted with caution, because brain MRI was performed in patients with less severe hemorrhagic severity. Third, the sample size in our study was small; therefore, the findings shown in this study need to be confirmed in future larger well-designed studies. Finally, the definition of recurrent ICH was based on index presentation and brain MRI characteristics but not on a follow-up period; thus, future follow-up studies investigating baseline CSVD burden and follow-up CSVD burden after ICH (i.e., one year after ICH onset among survival patients) are warranted to confirm these results.

## Conclusion

This study demonstrated that recurrent ICH occurred mostly among patients with mixed etiology and was associated with a higher CSVD burden than first-ever ICH. Additionally, the presence of ≥2 hematomas, mixed hematomas, and bilateral hematomas were separately correlated with a higher CSVD score. Future large studies are warranted to confirm our results.

## Supplemental data

Supplemental data are available at www.aginganddisease.org/EN/10.14336/AD.2017.0804.



## References

[1] Pantoni L, Fierini F, Poggesi A (2014). Thrombolysis in acute stroke patients with cerebral small vessel disease. Cerebrovasc Dis, 37:5-13. 24355873 10.1159/000356796

[2] Pantoni L (2010). Cerebral small vessel disease: from pathogenesis and clinical characteristics to therapeutic challenges. Lancet Neurol, 9:689-701. 20610345 10.1016/S1474-4422(10)70104-6

[3] van der Flier WM, van Straaten EC, Barkhof F, Verdelho A, Madureira S, Pantoni L, et al. (2005). Small vessel disease and general cognitive function in nondisabled elderly: the LADIS study. Stroke, 36:2116-2120. 16141425 10.1161/01.STR.0000179092.59909.42

[4] Herrmann LL, Le Masurier M, Ebmeier KP (2008). White matter hyperintensities in late life depression: a systematic review. J Neurol Neurosurg Psychiatry, 79:619-624. 17717021 10.1136/jnnp.2007.124651

[5] Gorelick PB, Scuteri A, Black SE, Decarli C, Greenberg SM, Iadecola C, et al. (2011). Vascular contributions to cognitive impairment and dementia: a statement for healthcare professionals from the american heart association/american stroke association. Stroke, 42:2672-2713. 21778438 10.1161/STR.0b013e3182299496 PMC3778669

[6] Wardlaw JM, Smith C, Dichgans M (2013). Mechanisms of sporadic cerebral small vessel disease: insights from neuroimaging. Lancet Neurol, 12:483-497. 23602162 10.1016/S1474-4422(13)70060-7 PMC3836247

[7] Roongpiboonsopit D, Charidimou A, William CM, Lauer A, Falcone GJ, Martinez-Ramirez S, et al. (2016). Cortical superficial siderosis predicts early recurrent lobar hemorrhage. Neurology, 87:1863-1870. 27694268 10.1212/WNL.0000000000003281 PMC5100711

[8] Klarenbeek P, van Oostenbrugge RJ, Rouhl RP, Knottnerus IL, Staals J (2013). Ambulatory blood pressure in patients with lacunar stroke: association with total MRI burden of cerebral small vessel disease. Stroke, 44:2995-2999. 23982717 10.1161/STROKEAHA.113.002545

[9] Staals J, Makin SD, Doubal FN, Dennis MS, Wardlaw JM (2014). Stroke subtype, vascular risk factors, and total MRI brain small-vessel disease burden. Neurology, 83:1228-1234. 25165388 10.1212/WNL.0000000000000837 PMC4180484

[10] Huijts M, Duits A, van Oostenbrugge RJ, Kroon AA, de Leeuw PW, Staals J (2013). Accumulation of MRI Markers of Cerebral Small Vessel Disease is Associated with Decreased Cognitive Function. A Study in First-Ever Lacunar Stroke and Hypertensive Patients. Front Aging Neurosci, 5:72. 24223555 10.3389/fnagi.2013.00072 PMC3818574

[11] Song TJ, Kim J, Song D, Yoo J, Lee HS, Kim YJ, et al. (2017). Total Cerebral Small-Vessel Disease Score is Associated with Mortality during Follow-Up after Acute Ischemic Stroke. J Clin Neurol, 13:187-195. 28406586 10.3988/jcn.2017.13.2.187 PMC5392462

[12] Uiterwijk R, van Oostenbrugge RJ, Huijts M, De Leeuw PW, Kroon AA, Staals J (2016). Total Cerebral Small Vessel Disease MRI Score Is Associated with Cognitive Decline in Executive Function in Patients with Hypertension. Front Aging Neurosci, 8:301. 28018214 10.3389/fnagi.2016.00301 PMC5149514

[13] Wardlaw JM, Smith EE, Biessels GJ, Cordonnier C, Fazekas F, Frayne R, et al. (2013). Neuroimaging standards for research into small vessel disease and its contribution to ageing and neurodegeneration. Lancet Neurol, 12:822-838. 23867200 10.1016/S1474-4422(13)70124-8 PMC3714437

[14] Lau KK, Li L, Schulz U, Simoni M, Chan KH, Ho SL, et al. (2017). Total small vessel disease score and risk of recurrent stroke: Validation in 2 large cohorts. Neurology, 88:2260-2267. 28515266 10.1212/WNL.0000000000004042 PMC5567324

[15] Charidimou A, Imaizumi T, Moulin S, Biffi A, Samarasekera N, Yakushiji Y, et al. (2017). Brain hemorrhage recurrence, small vessel disease type, and cerebral microbleeds: A meta-analysis. Neurology, 89:820-829. 28747441 10.1212/WNL.0000000000004259 PMC5580863

[16] Charidimou A, Werring DJ (2014). Cerebral microbleeds as a predictor of macrobleeds: what is the evidence? Int J Stroke, 9:457-459. 24798040 10.1111/ijs.12280

[17] Linn J, Halpin A, Demaerel P, Ruhland J, Giese AD, Dichgans M, et al. (2010). Prevalence of superficial siderosis in patients with cerebral amyloid angiopathy. Neurology, 74:1346-1350. 20421578 10.1212/WNL.0b013e3181dad605 PMC2875936

[18] Charidimou A, Boulouis G, Haley K, Auriel E, van Etten ES, Fotiadis P, et al. (2016). White matter hyperintensity patterns in cerebral amyloid angiopathy and hypertensive arteriopathy. Neurology, 86:505-511. 26747886 10.1212/WNL.0000000000002362 PMC4753727

[19] Pasi M, Boulouis G, Fotiadis P, Auriel E, Charidimou A, Haley K, et al. (2017). Distribution of lacunes in cerebral amyloid angiopathy and hypertensive small vessel disease. Neurology, 88:2162-2168. 28476760 10.1212/WNL.0000000000004007 PMC5467956

[20] Wu B, Yao X, Lei C, Liu M, Selim MH (2015). Enlarged perivascular spaces and small diffusion-weighted lesions in intracerebral hemorrhage. Neurology, 85:2045-2052. 26546632 10.1212/WNL.0000000000002169 PMC4676754

[21] Greenberg SM, Vernooij MW, Cordonnier C, Viswanathan A, Al-Shahi Salman R, Warach S, et al. (2009). Cerebral microbleeds: a guide to detection and interpretation. Lancet Neurol, 8:165-174. 19161908 10.1016/S1474-4422(09)70013-4 PMC3414436

[22] Charidimou A, Boulouis G, Xiong L, Jessel MJ, Roongpiboonsopit D, Ayres A, et al. (2017). Cortical superficial siderosis and first-ever cerebral hemorrhage in cerebral amyloid angiopathy. Neurology, 88:1607-1614. 28356458 10.1212/WNL.0000000000003866 PMC5405764

[23] Fazekas F, Chawluk JB, Alavi A, Hurtig HI, Zimmerman RA (1987). MR signal abnormalities at 1.5 T in Alzheimer’s dementia and normal aging. AJR Am J Roentgenol, 149:351-356. 3496763 10.2214/ajr.149.2.351

[24] Charidimou A, Meegahage R, Fox Z, Peeters A, Vandermeeren Y, Laloux P, et al. (2013). Enlarged perivascular spaces as a marker of underlying arteriopathy in intracerebral haemorrhage: a multicentre MRI cohort study. J Neurol Neurosurg Psychiatry, 84:624-629. 23412074 10.1136/jnnp-2012-304434 PMC3905629

[25] Imaizumi T, Inamura S, Nomura T (2014). The severities of white matter lesions possibly influence the recurrences of several stroke types. J Stroke Cerebrovasc Dis, 23:1897-1902. 24784013 10.1016/j.jstrokecerebrovasdis.2014.02.011

[26] Pasi M, Charidimou A, Boulouis G, Auriel E, Ayres A, Schwab KM, et al. (2018). Mixed-location cerebral hemorrhage/microbleeds: Underlying microangiopathy and recurrence risk. Neurology, 90:e119-e126. 29247070 10.1212/WNL.0000000000004797 PMC5772153

