# Supplementary material for: Total Burden of Cerebral Small Vessel Disease in Recurrent ICH versus First-ever ICH
Source: Aging Dis. 2019 Jun 1;10(3):570–7. doi: 10.14336/AD.2018.0804 (PMC6538213; doi:10.14336/AD.2018.0804)
Supplement: Supplementary file 1 [file AD-10-3-570-s.pdf]

SUPPLEMENTARY DATA

Original Article

Total Burden of Cerebral Small Vessel Disease in Recurrent ICH versus First-ever ICH

Mangmang Xu, Yajun Cheng, Quhong Song, Ruozhen Yuan, Shuting Zhang, Zilong Hao, Ming Liu

Center of Cerebrovascular Disease, Department of Neurology, West China Hospital, Sichuan University. Chengdu, 610041, Sichuan Province, China

© 2018. Xu M et al. Published online at http://www.aginganddisease.org/EN/10.14336/AD.2018.0804

SUPPLEMENTARY DATA

Supplemental Table 1. Kappa value of the neuroimaging characteristics

Variable	Kappa value
The presence of lacunes	0.783
The presence of CMBs	1.000
Strictly deep CMBs	0.857
Strictly lobar CMBs	0.643
Mixed CMBs	0.800
cSS	0.737
WMH	1.000
BG EPVS>10	0.700
Mixed hematomas	0.765
History of ICH	0.875
Bilateral hematomas	0.898
≥2 hematomas	0.783

Abbreviation: CMB, cerebral microbleed; WMH, white matter hyperintensities; EPVS, enlarged perivascular spaces; BG, basal ganglia; ICH, intracerebral hemorrhage; cSS, cortical superficial siderosis.

Supplemental Table 2. The severity of WMH in HA-, CAA-, and mixed etiology-ICH.

	HA (n=75)	CAA (n=26)	Mixed etiology (n=57)	*p* value
The presence of WMH, n (%)	19 (25.3)	15 (57.7)	45 (78.9)	p<0.00000001
The presence of deep WMH 2-3, n (%)	16 (21.3)	13 (50.0)	42 (73.7)	p<0.0000001
The presence of periventricular WMH 3, n (%)	11 (14.7)	7 (26.9)	31 (54.4)	p<0.00001

The ICH etiology in 26 patients was undetermined, so 158 patients were included into the analysis.

Supplemental Table 3. Clinical characteristics between primary ICH patients with and without MRI (including SWI).

Variables	Primary ICH with MRI (n=184)	Primary ICH without MRI (n=1033)	*p* value
Age, Y, mean (SD)	61.0 (12.5)	57.5 (13.8)	0.002
History of hypertension, n (%)	122 (66.3)	882 (85.4)	<0.001
History of DM, n (%)	17 (9.2)	83 (8.0)	0.561
History of hyperlipidemia, n (%)	6 (3.3)	13 (1.3)	0.054
Smoking, n (%)	61 (33.2)	242 (23.4)	0.005
The severity of stroke			
GCS, median (IQR)	15 (12-15)	12 (7-15)	<0.001
NIHSS, median (IQR)	6 (2-9)	9 (4-18)	<0.001
ICH location, n (%)			
Deep	128 (69.6)	715 (69.2)	0.925
Lobar	48 (26.1)	259 (25.1)	0.770
Cerebellum	8 (4.3)	60 (5.8)	0.427

*One patient among the 1033 primary ICHs without MRI had hematoma located in both lobar and deep.
